# Hole Polaron Migration in Bulk Phases of TiO_2_ Using Hybrid Density Functional Theory

**DOI:** 10.1021/acs.jpcc.1c03136

**Published:** 2021-05-27

**Authors:** John J. Carey, James A. Quirk, Keith P. McKenna

**Affiliations:** Physics Department, University of York, York, Heslington YO10 5DD, U.K.

## Abstract

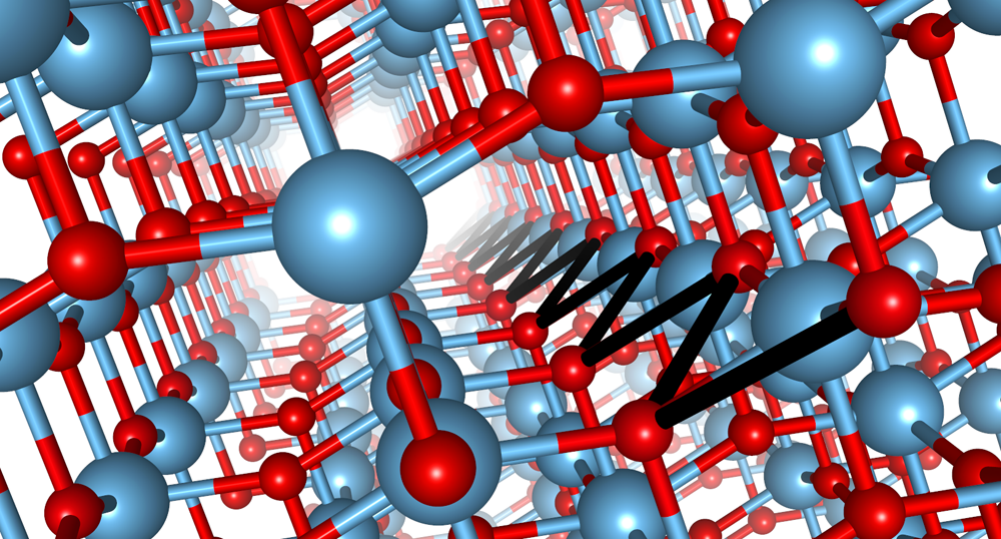

Understanding charge-carrier transport in semiconductors is vital to the improvement of material performance for various applications in optoelectronics and photochemistry. Here, we use hybrid density functional theory to model small hole polaron transport in the anatase, brookite, and TiO_2_-B phases of titanium dioxide and determine the rates of site-to-site hopping as well as thermal ionization into the valance band and retrapping. We find that the hole polaron mobility increases in the order TiO_2_-B < anatase < brookite and there are distinct differences in the character of hole polaron migration in each phase. As well as having fundamental interest, these results have implications for applications of TiO_2_ in photocatalysis and photoelectrochemistry, which we discuss.

## 1 Introduction

Titanium dioxide (TiO_2_) is a non-toxic and earth-abundant semiconductor that finds a number of photocatalytic and renewable energy applications, for example, as an electron transport layer in solar cell devices,^1^ a photocatalyst for removing harmful NO_*x*_ species from the environment, and a water splitting catalyst for hydrogen production.^2^ TiO_2_ has a number of polymorphs with anatase, rutile, brookite, and TiO_2_-B being the best studied. A wide variety of nanostructures including nanocrystals of different shapes, nanorods, and nanosheets are also commonly explored, offering additional parameters for material optimization. Many of the applications above rely directly or indirectly on the efficient transport of charge carriers through the material. For example, for photochemical applications, electrons and holes that are photogenerated in the bulk need to diffuse efficiently to surfaces where they can interact with adsorbates to facilitate chemical reactions.^3,4^ For efficient solar cell devices, electrons must diffuse effectively through nanoporous titania layers to the electrode interfaces.^5^ The strong electron–phonon coupling in TiO_2_ means that in many cases, photogenerated electrons and holes can self-trap to form small polarons (quasiparticles consisting of a charge carrier and an associated lattice deformation).^6,7^ Since the mobility of small polarons is much lower than that of band-like charge carriers (or large polarons) such self-trapping can enhance rates of recombination (radiative and non-radiative), negatively affecting material performance for applications.^8−10^ However, the dominant mechanisms of charge transport in TiO_2_ are still not well understood. Even if small polarons can form, it does not necessarily mean the mechanism of long range carrier diffusion is purely polaronic (i.e., hopping of polarons between neighboring lattice sites). Instead, it may be more favorable for the small polaron to thermally ionize before propagating as a band-like carrier and subsequently retrapping.^11−13^ It is challenging to probe such processes experimentally and a deep understanding is currently missing. Aside from its fundamental importance, atomistic insights into these effects could ultimately help in the development of strategies to control and improve charge-carrier mobility for applications.

In this article, we tackle this problem theoretically and investigate the mobility of holes in the three common phases of TiO_2_ where hole polarons are predicted to be stable (anatase, brookite, and TiO_2_-B). This is a challenging problem since it requires approximations to the many-body problem that are sufficiently accurate to correctly describe the crystal and electronic structures as well as the stability of localized versus delocalized charge carriers. Here, we use a hybrid density functional theory (DFT) approach, which has been explicitly parameterized for accurate description of small polarons in TiO_2_ using a known property of exact DFT.^14^ Using this approach, single site small polaron hole localization is favorable only in anatase, brookite, and TiO_2_-B and so we focus on these three polymorphs for simulation of hole mobility. Hole trapping in rutile on the other hand is not predicted to be favorable. Multisite (or molecular) hole polarons are also predicted to be stable in TiO_2_-H and TiO_2_-R but are not considered here since they are less relevant experimentally and hopping of multisite polarons would be considerably more complex to model. Our key findings are that long-range diffusion of holes in brookite proceeds via thermal ionization and band-like conduction since the thermal ionization energy of the small hole polaron (0.12 eV) is small compared to the activation energies for polaron hopping. The thermal ionization energy for small hole polarons in anatase is larger (0.24 eV) and therefore at low temperatures, holes diffuse by polaronic hopping, with preferential diffusion in the [100] and [010] directions, but at room temperature the migration has a mixed character. TiO_2_-B has the largest ionization energy (0.53 eV), suggesting polaronic hole hopping to be more dominant. The anisotropy in polaron mobility and differences between the three phases have important implications for understanding and designing materials for applications, which we discuss.

The remainder of the article is organized in the following way. In Section 2, we discuss the importance of hole polarons TiO_2_ with respect to previous experimental studies and applications. We also highlight the challenges associated with accurate modeling of small polarons and discuss previous theoretical studies on TiO_2_. In Section 3, we present the computational methods employed before presenting the results in Section 4. Finally, we present a detailed discussion and our conclusions.

## 2 Background

TiO_2_ is usually intrinsically n-type due to the presence of oxygen vacancies or titanium interstitials, which act as shallow donors. However, holes can be generated on photoexcitation and their subsequent dynamics is important for understanding a number of processes of fundamental and practical significance. For example, the migration of photoinduced holes through the lattice to surfaces is essential for photocatalysis.^15^ A number of studies have highlighted facet-dependent reactivity in nanocrystal systems, which has been interpreted in terms of the different stabilities of hole polarons on different surfaces.^16,17^ Although relatively unexplored, anisotropy in hole diffusion may also play a role. The dynamics of photoinduced holes are critical in nanostructured TiO_2_ photoelectrodes for water splitting.^18,19^ Complex features in temperature-dependent photoluminescence spectra can also be interpreted in terms of carrier dynamics, where evidence suggests that in general holes are less mobile than electrons and the determining factor for recombination.^20,21^ The examples given above provide clear evidence of the importance of hole polaron migration in TiO_2_ and need for deeper atomistic insights.

There have been numerous DFT investigations into the stability and electronic properties of hole polarons in TiO_2_ in perfect and amorphous crystals as well as at extended defects.^8,9,14,22−34^ Standard local or semi-local exchange correlation functionals fail to describe hole polarons correctly due to significant residual self-interaction error (SIE), which tends to favor delocalized electronic states. Therefore, most of the above studies have been performed using modifications to standard DFT such as DFT + *U* or hybrid functionals incorporating some proportion of Hartree–Fock (HF) exchange (hybrid DFT). Predicted polaron properties are sensitive to the parameterization of these methods (i.e., the *U* in DFT + *U* or the fraction of HF exchange), with some methods overlocalizing and others underlocalizing. In principle, the generalized Koopmans condition (a known property of the exact functional) provides a means to constrain the choice of parameters in the functional, ensuring a more complete cancellation of the SIE^35−38^ It has been recently shown for model systems in 1D (for which the many-body problem can be solved exactly) that hybrid functionals parameterized in this way provide ionization energies and electron densities in excellent agreement with exact solutions.^39^ This approach has been employed to predict the properties of stable electrons and hole polarons in all known phases of TiO_2_, where the use of large supercells (>500 atoms) also minimizes finite size effects as well as at surfaces, twin boundaries, and nanocrystals.^14,31,40−42^

DFT calculations of polaron hopping in TiO_2_ are less common. A few studies using the Marcus–Emin–Holstein–Austin–Mott (MEHAM) formalism^6,43−45^ together with DFT + *U* have explored electron and hole polaron migration in anatase and rutile.^22^ For holes in anatase, hopping is predicted to be non-adiabatic for most intersite pathways with activation energies in the range 0.17–0.59 eV.^26^ However, the size of the SIE in these calculations is unknown (and could be significant given the large Hubbard *U* value used of 10 eV). Although hybrid functional methods have been used together with MEHAM theory to investigate electron transfer process in some materials, for example, MgO and HfO_2_,^46−48^ it is yet to be applied to bulk phases of TiO_2_ (including brookite or TiO_2_-B). Furthermore, previous studies have not compared activation energies for thermal ionization of the polaron against activation energies for hole hopping at the same level of theory which is needed in order to determine the migration mechanism.

## 3 Methods

Hybrid DFT calculations using the generalized gradient approximation were carried out using the CP2K simulation package.^49^ Exact HF exchange is mixed into the exchange–correlation functional to overcome the issue of the SIE that is well known in DFT. We use a truncated PBE0 hybrid-DFT exchange–correlation functional that includes long-range corrections to the interaction potential (PBE0-TR-LRC) with a global 1/*r* dependence.^49^ This defines a range of separations in the electron integrals to implement the HF exact exchange, and standard PBE is used outside this defined range. The truncation radius (*R*_c_) must be less than half the length of the smallest supercell lattice vector to ensure that there is no interaction between neighboring cells, and we set our radius to 6.0 Å, shown previously to give converged structural and electrical properties.^14^ The percentage of HF exact exchange (α) to include in these calculations was stringently parameterized by satisfying Koopmans’ condition with a 0.05 eV tolerance for electron and hole polarons in each of the bulk TiO_2_ phases, providing a band gap within 3% of the experimental value.^14^ This was achieved by first optimizing the bulk crystal structure and hole polaron geometry for different choices of α. Then, for each case, the charge transition energy associated with adding an electron vertically (without geometry optimization) is computed and compared with the corresponding highest occupied eigenvalue. To satisfy Koopmans’ condition (a necessary requirement for an exact functional), these two energies should be equal, providing a means to determine optimal values for each phase. We refer the reader to ref (14) where full details on this approach are provided. The optimal α for each phase is: 11.5% for anatase, 10.5% for brookite, and 12% for TiO_2_-B. Triple ζ basis sets were used for both titanium and oxygen for accurate calculations^50,51^ and the Goedecker–Teter–Hutter (GTH) pseudopotentials for both species available within CP2K.^52−54^ A multi-grid approach for mapping products of Gaussians onto a real-space integration grid is used in CP2K, where the wide and smooth Gaussian functions are mapped onto a coarser grid, and the electron density is mapped onto the finest grid. The plane wave energy cut-off, a reference grid which controls the Gaussian mapping onto the multi-grid, is set to 60 Ry. Five multi-grids are used, and the plane wave cut-off is sufficiently converged at 600 Ry for the finest level of the multi-grid. The cell vectors and bulk geometries were optimized using the Broyden–Fletcher–Goldfarb–Shanno method where the forces were converged to less than 8 × 10^–4^ Ry/a_0_ (0.02 eV/Å) and the electronic convergence was set to 1 × 10^–6^ Ry per self-consistent field (SCF) cycle.

Our previous work has shown that small hole polarons can form in anatase, brookite, and TiO_2_-B, and the calculated bulk structures and corresponding hole polaron geometries are taken as starting points for this investigation, see Elmaslmane and McKenna for computational details.^14^ The supercells we use are approximately cubic in shape and contain 600 (anatase), 576 (brookite), and 720 (TiO_2_-B) atoms with the length of lattice constants in the range 16–25 Å. Since only the hole is introduced to the system without an accompanying electron, finite size effects from the electrostatic interaction of the hole with periodic images are present. However, we have performed careful supercell scaling calculations in our previous work and shown that the large supercells we employ are sufficient to minimize finite size effects.^14^ Furthermore, calculation of barriers to hole hopping are far less affected by finite size effects since the hole remains localized throughout transition. To model polaron hopping, we employ the MEHAM model, which is able to describe the charge-transfer processes in both the adiabatic and non-adiabatic limits and is well suited to describing small polaron hopping in TiO_2_.^6,43−45^ There are many previous applications of the approach to model polaron hopping in TiO_2_, Fe_2_O_3_, and organic semiconductors as well as electron transfer between point defects in crystals.^22,26,46−48,55,56^ Here, we employ MEHAM with the common approximation that the hole hopping process can be described as a one-dimensional process coupling to a single generalized phonon mode. We note that although we consider a single phonon mode, it includes displacements of all atoms and the wavefunction is not constrained in any way, so it is not the case that adjacent atoms are not involved (in general they all are). The reasonableness of the one-dimensional approximation is discussed in detail in the work by Alkauskas et al.^57^ and references therein. By considering such hopping processes between all inequivalent sites, we can describe hopping via adjacent atoms by chaining together these individual transitions we discuss in Section 4.3.

**Figure 1:**
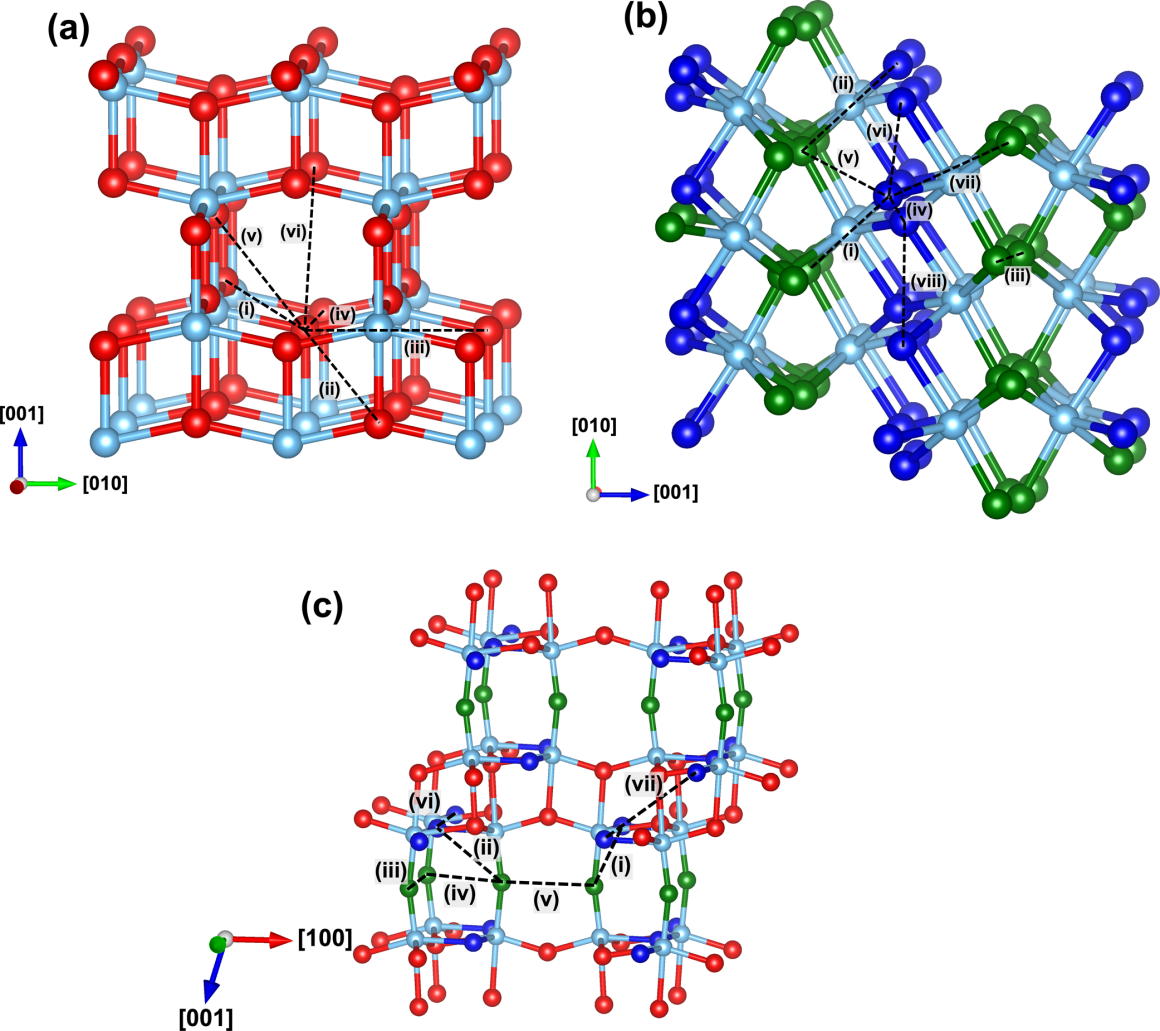
Optimized bulk structures for (a) anatase, (b) brookite, and (c) TiO_2_-B. Possible pathways for hopping of small hole polarons are shown and labeled using Roman numerals. For brookite and TiO_2_-B, there are two inequivalent oxygen sites that can trap holes. The most stable site is indicated by the blue spheres and second most stable by the green spheres. Note that for TiO_2_-B there are some sites that do not trap holes at all (red spheres).

For each of the TiO_2_ phases, we consider the hopping of small polarons between different anion sites along various pathways as indicated in Figure 1. These pathways include all paths with inter-anion separations of less than 4 Å. With the pathways identified, the first step is to obtain the optimized geometries for the polaron localized on both the initial and final anion sites (denoted by the set of coordinates **R**_*i*_^a^ and **R**_*i*_^b^, respectively, where *i* is an index labeling each atom). Atomic structures for hole polarons localized on different sites are obtained by introducing a precursor potential well for polaron trapping by displacing neighboring Ti atoms around a given O site by 0.3 Å followed by full geometry optimization. As shown recently in a separate study, this approach is highly efficient for obtaining ground state structures of polarons.^58^ We then linearly interpolate between the initial and final geometries to define a one-dimensional reaction coordinate for polaron hopping
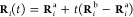
1

The corresponding one-dimensional potential energy surface (PES) is then computed as *E*[**R**(*t*)]. One can calculate diabatic PESs (where the hole remains localized on one site or the other as *t* varies) by initializing calculations using the converged wavefunction from the adjacent point in the pathway (starting from *t* = 0 and increasing as well as starting at *t* = 1 and decreasing). The adiabatic PES (where the hole is delocalized across the sites) can be computed by performing the same calculation with random initialization of the wavefunction. From these PESs, we are able to compute or estimate the various parameters needed to evaluate the rate of polaron hopping using MEHAM theory, including the adiabatic activation energies, the tunneling matrix elements, and the effective one-dimensional phonon-mode frequency coupling to the charge-transfer process (details given in the following section). We note that it is important to have well controlled self-interaction errors to accurately calculate the PESs required. In the next section, we present results for various polaron hopping pathways in each of the bulk phases of titania as well detail the approach to determine the various parameters used to calculated the polaron migration rates. All structural images and spin density plots are visualized using the VESTA software.^59,60^

## 4 Results

### 4.1 Polaron Migration in Anatase, Brookite, and TiO_2_-B

The optimized bulk structures of anatase, brookite, and TiO_2_-B are shown in Figure 1. In anatase, all anion sites are equivalent, with oxygen atoms coordinated to three titanium atoms in a trigonal planar geometry. The hole small polaron in anatase is predicted to localize primarily onto a single oxygen atom. In brookite, there are two inequivalent oxygen sites in the bulk structure. The more stable O_1_ site and less stable O_2_ (difference in energy 0.03 eV) are indicated by blue and green spheres, respectively in Figure 1b. Similar to anatase, both oxygen sites are coordinated to three titanium atoms in a trigonal planar geometry. Of the four inequivalent oxygen sites in the TiO_2_-B phase, small hole polarons are found to localize on only two of them. The more stable O_1_ site and less stable O_2_ (difference in energy 0.11 eV) are indicated by blue and green spheres, respectively, in Figure 1c. Site O_1_ again adopts a three-coordinated trigonal planar geometry, while O_2_ is coordinated to two titanium atoms in a linear configuration.

**Figure 2:**
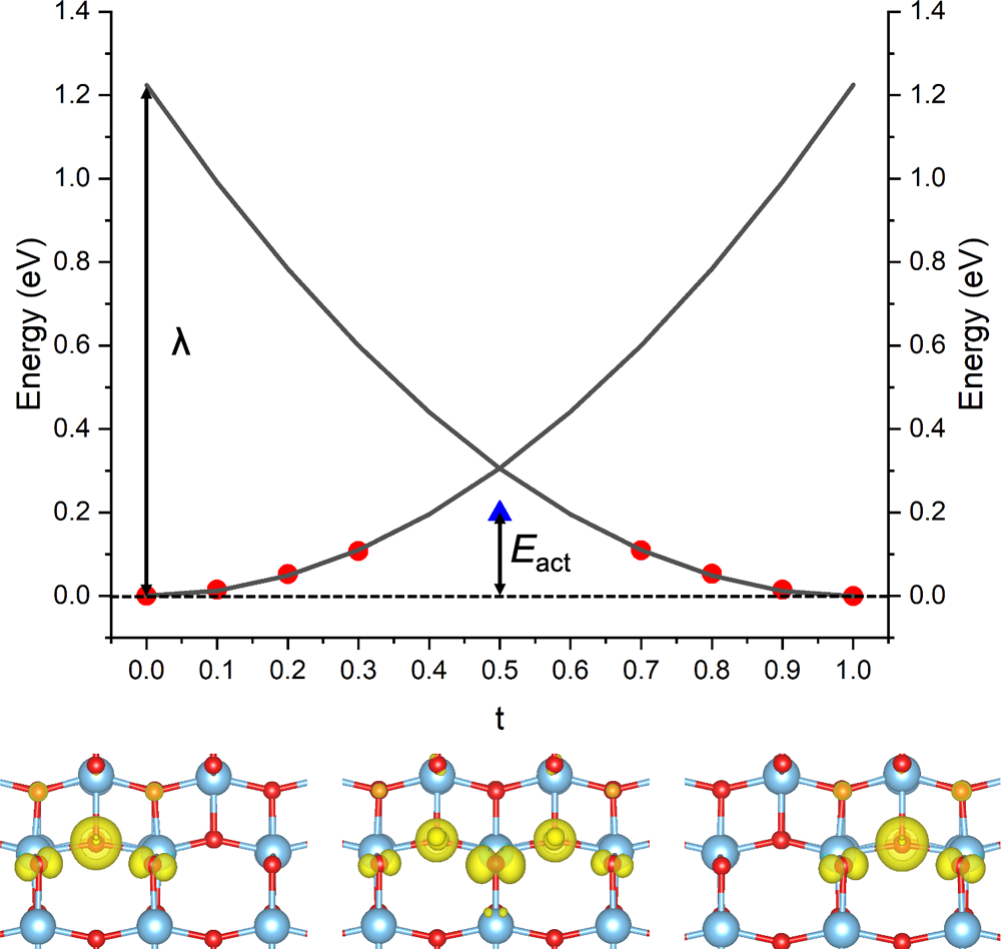
(a) Diabatic PES for the hole polaron hopping pathway (ii) in anatase. The fits for the diabatic curves are shown as black lines, while the adiabatic energy at the transition state is shown by the blue triangle. The localized polaron spin density for the end points and adiabatic solution at the transition state are shown below. The reorganization energy (λ) and activation energy (*E*_act_) are indicated.

For all of the pathways identified in Figure 1, we perform linear interpolations to obtain the PESs for small polaron hopping (as described in Methods and eq 1). For each pathway, we calculate the total energy at intervals Δ*t* = 0.1. To illustrate our approach, we show a concrete example in Figure 2 that corresponds to the hole hopping pathway (ii) in anatase (see Figure 1a). The spin density is also shown in the figure highlighting that at the transition state (*t* = 0.5), the hole polaron is symmetrically localized across the two sites, corresponding to an adiabatic solution. The adiabatic activation energy (*E*_act_) can therefore be determined directly from this PES. Initially, we attempted to also compute the diabatic PES at the transition state (*t* = 0.5) by carefully initializing the calculation with a charge density corresponding to hole localization on one of the sites only. However, while we were able to obtain symmetry broken solutions, there was still a significant adiabatic character in the solution (i.e., partial delocalization over the two sites). As an alternative, we computed an estimate of the diabatic energy at the transition state by fitting a harmonic function to the points *t* < 0.4 where the spin density shows a diabatic-like solution (black curves in Figure 2a).

The fitted diabatic PESs provide estimates for the diabatic energy *E*_dia_ at *t* = 0.5, the reorganization energy λ at *t* = 1, and the effective phonon frequency (Ω) corresponding to this one-dimensional electron transfer model
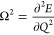
2where *Q* is the generalized configuration coordinate
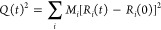
3and *M*_*i*_ are the atomic masses. The electronic coupling constant *H*_ab_ is then estimated as the difference between the adiabatic and diabatic energies at the transition state. We note a more accurate approach would be to obtain the diabatic solutions at the transition state using constrained DFT and then diagonalizing to obtain the electronic coupling constant as we have done in previous studies.^46−48^ However, the high computational cost of these calculations and large number of pathways make this prohibitive at the present time.

With the various parameters calculated or estimated above, we proceed to calculate the polaron migration rate (*k*_et_) using the MEHAM theory as follows
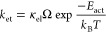
4where the electronic transmission coefficient is
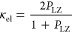
5*P*_LZ_ is the Landau–Zener transition probability
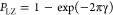
6and γ is the adiabaticity parameter defined as
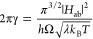
7

Table 1 shows the calculated parameters for pathway (ii) in anatase together with those of the other pathways in anatase and the other phases considered. The calculated adiabatic activation energy for pathway (ii) is 0.20 eV with *H*_ab_ = 109 meV. The pathways for each phase in Figure 1 are labeled according to the corresponding activation energies from smallest to largest. Pathway (i) with the smallest activation energy (0.12 eV) and reorganization energy (0.93 eV) in anatase corresponds to hopping between neighboring oxygen sites separated by 2.80 Å. The calculated effective phonon frequencies (Ω) are also found to be consistent with typical infrared active modes measured experimentally in anatase (e.g., the *E*_u_(1) mode at 358 cm^–1^^61,62^).

**Table 1:** Distance between Oxygen Sites in the Ideal Crystal (*d*_O–O_), Adiabatic Activation Energy (*E*_act_), Reorganization Energy (λ), Electronic Coupling Matrix (*H*_ab_), Difference in Energy between the Initial and Final Equilibrium Configurations (Δ*G*^0^), Effective Optical Phonon Frequency for the Electron Transfer Process (Ω), and the Calculated Polaron Hopping Rate (*k*_et_) at Room Temperature (300 K) for Various Pathways in Each of the Titania Phases^a^

pathway	*d*_O–O_ (Å)	*E*_act_ (eV)	*H*_ab_ (meV)	λ (eV)	Δ*G*^0^ (eV)	Ω (cm^–1^)	*k*_et_ [300 K] (Hz)
Anatase							
i	2.80	0.12	113	0.93	0.00	285	8.43 × 10^10^
ii	2.47	0.20	109	1.23	0.00	320	4.69 × 10^9^
iii	3.80	0.22	134	1.41	0.00	335	2.18 × 10^9^
iv	3.81	0.29	125	1.64	0.00	359	1.74 × 10^8^
v	3.08	0.33	40	1.46	0.00	348	2.73 × 10^7^
vi	3.74	0.36	48	1.62	0.00	331	8.67 × 10^6^
Brookite							
i	2.72	0.08	75	0.76	–0.03	210	2.09 × 10^11^/7.63 × 10^10^
ii	2.73	0.09	100	0.77	–0.03	202	1.74 × 10^11^/7.52 × 10^10^
iii	2.76	0.14	106	0.97	0.00	220	3.20 × 10^10^
iv	2.82	0.15	90	0.96	0.00	247	2.28 × 10^10^
v	2.53	0.23	80	1.37	–0.03	247	7.29 × 10^8^/2.98 × 10^8^
vi	2.50	0.24	74	1.27	0.00	257	6.07 × 10^8^
vii	3.86	0.29	80	1.58	–0.03	329	1.53 × 10^8^/4.94 × 10^7^
viii	2.81	0.33	4	1.35	0.00	277	6.77 × 10^5^
TiO_2_-B							
i	2.71	0.04	144	0.72	–0.11	236	1.74 × 10^12^/2.30 × 10^10^
ii	2.87	0.23	151	1.63	–0.11	327	1.30 × 10^9^/1.78 × 10^7^
iii	3.72	0.24	140	1.52	0.00	321	8.77 × 10^8^
iv	3.45	0.38	81	1.83	0.00	337	4.87 × 10^6^
v	3.23	0.39	88	1.90	0.00	337	3.25 × 10^6^
vi	3.74	0.39	78	1.87	0.00	364	3.20 × 10^6^
vii	4.76	0.41	69	1.91	0.00	345	1.38 × 10^6^

a For asymmetric paths, the average for λ and Ω is given along with the rate in both directions. See Figure 1 for definition of the pathways.

**Figure 3:**
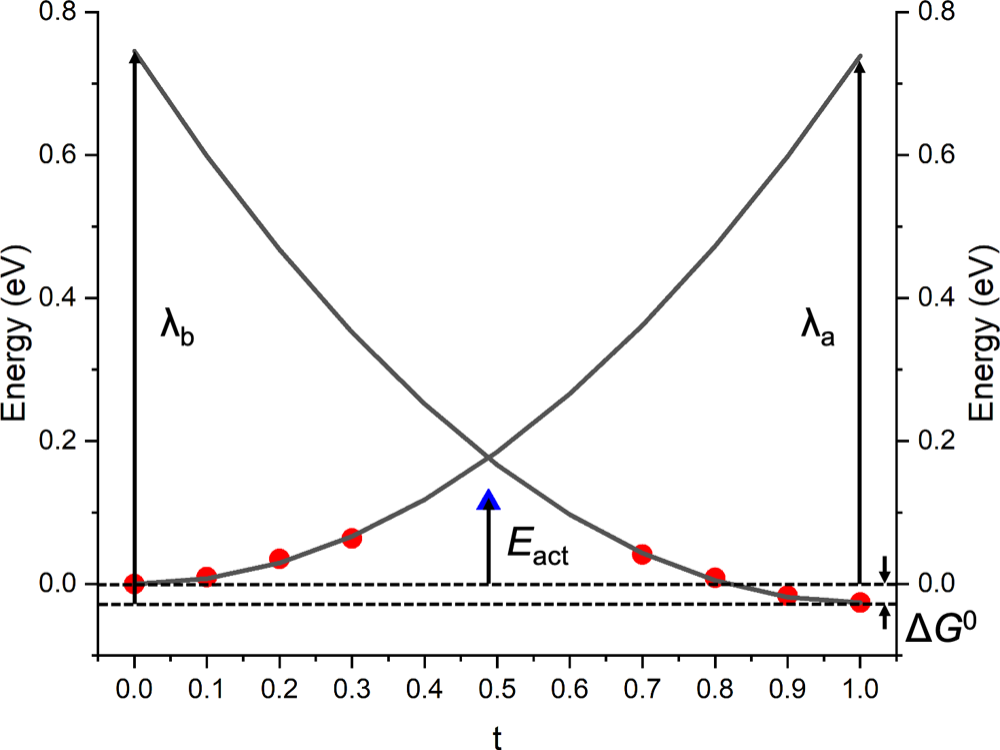
Calculated diabatic PES and adiabatic energy at the transition state for an asymmetric polaron pathway in brookite [pathway (i)]. The reorganization energies for the left and right diabatic curves (λ_a_) and (λ_b_), the activation energy (*E*_act_), and the difference in energy between the two energy minima (Δ*G*^0^) are indicated.

Turning to brookite, the situation becomes more complex since there are two inequivalent oxygen sites and more pathways to consider. In the cases of hopping between inequivalent oxygen sites, the PES for hole hopping becomes asymmetric. For example, Figure 3a shows the calculated PES for pathway (i) in brookite. The energy difference between the two end points (Δ*G*^0^) must be taken into account when calculating the hole hopping transfer rate using MEHAM theory.^26,63^ In the following, we consider hole hopping from the higher to the lower energy site. The activation energy for hole hopping in the reverse direction is then simply given by *E*_act_ – Δ*G*^0^. The transition state for asymmetric pathways does not occur at *t* = 0.5; hence, we need to account for this in the determination of *E*_act_ by evaluating the adiabatic energy at the transition state once the fits for the diabatic PESs have been performed. However, in all cases considered, the transition state is within 0.014 (brookite) and 0.023 (TiO_2_-B) of 0.5 and so the adiabatic energy at the mid-point is a very good approximation. In general, for asymmetric pathways, the diabatic PESs also have different reorganization energies and vibrational frequencies. For simplicity, here we simply take an average of these quantities to evaluate κ_el_, which should be a reasonable approximation since these values do not differ significantly.

The brookite structure consists of alternating (001) oriented layers of O_1_ and O_2_ oxygen sites as shown in Figure 2b. Therefore, hole migration in the [100] and [010] directions involves hopping between equivalent oxygen sites, while migration in the [001] direction involves hopping between inequivalent sites. Table 1 shows the calculated hole hopping parameters for all pathways in brookite. The lowest energy pathway (i) for the hole migration in brookite is along the [001] direction between two different O sites with an activation energy of 0.08 eV (or 0.13 eV in the reverse direction). Direct hopping between the most stable O_1_ sites—pathways (iv) and (vi)—are associated with higher activation energies of 0.15 and 0.24 eV. The calculated effective phonon frequencies are again in good agreement with experimental data.^64^

The structure for the TiO_2_-B phase is much more complicated than either anatase or brookite since there are four inequivalent O sites; however, hole polarons localize on only two of them, with three-coordinated site O_1_ more stable than the two-coordinated site O_2_ by 0.11 eV (Figure 1c). For the other two sites, delocalized holes are more favorable than the localized holes for the value of α that satisfies Koopmans’ condition. The calculated hole hopping parameters for all pathways in TiO_2_-B are shown in Table 1. The most favorable pathway (i) involves hopping from site O_2_ to site O_1_ with an activation energy of 0.04 eV (0.15 eV in the reverse direction). Direct hopping between O_1_ sites is possible via only two pathways: pathway (vi) in the [010] direction with an activation energy of 0.39 eV and pathway (vii) with an activation energy of 0.41 eV. These activation energies are sufficiently high that long-range diffusion in TiO_2_-B is likely to proceed via the less stable O_2_ sites (discussed further below). Finally, we note that all of the pathways in Table 1 are adiabatic (κ_el_ ∼ 1) except for brookite pathway (viii) which as discussed below is in practice not accessible in any case.

### 4.2 Thermal Ionization of Hole Polarons in Anatase, Brookite, and TiO_2_-B

**Figure 4:**
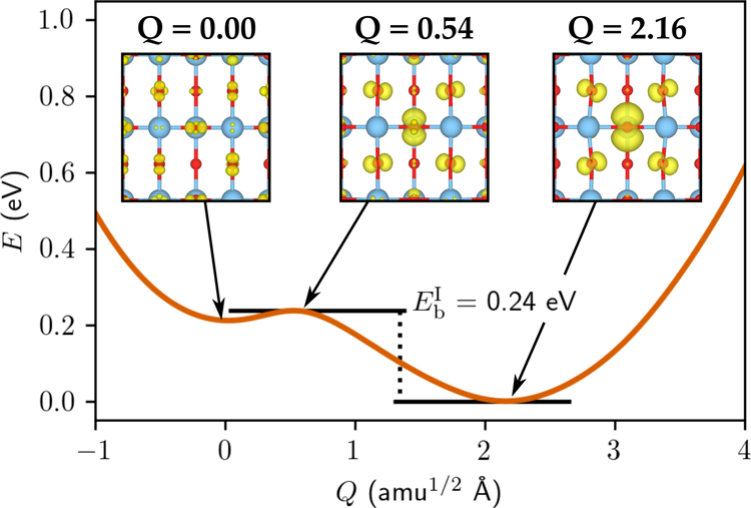
1D configuration-coordinate diagram for the anatase hole polaron in the positive charge state. *Q* = 0.00 amu^1/2^ Å corresponds to the bulk geometry and *Q* = 2.16 amu^1/2^ Å corresponds to the polaron geometry. The insets show corresponding spin density isosurfaces.

To compute the energy barriers to thermal ionization of polarons into the valence band, *E*_b_^I^, we compute the corresponding adiabatic PES by linearly interpolating between the polaron geometry and the bulk geometry, defining a corresponding configuration-coordinate, *Q* (where *Q* = 0 is the geometry of the ideal bulk crystal and *Q* = Δ*Q* is the equilibrium geometry of the polaron). The energy was evaluated between the range −Δ*Q* to 2Δ*Q* (to capture the local minima at *Q* = 0 and *Q* = Δ*Q* as well as the curvature on either side) with a spacing of 0.1Δ*Q* between points. From these PESs, we compute *E*_b_^I^ (which is the barrier to cross from the minima at *Q* = Δ*Q* to the minima at *Q* = 0) and the barrier to self-trapping *E*_b_^ST^, which is the barrier for the reverse process (where one exists). Figure 4 shows the computed PES for anatase, where *E*_b_^I^ = 0.24 eV and *E*_b_^ST^ = 0.03 eV. The calculated values for the ionization and trapping barriers for the other phases and hole polaron sites are given in Table 2. For all phases of TiO_2_, the barrier to trapping is much lower than the barrier to ionization, and in the case of TiO_2_-B, there is no barrier at all for trapping. The ionization barrier is relatively large for TiO_2_-B, suggesting that the polarons are highly stable for these phases, while for brookite the ionization barriers are much lower.

**Table 2:** Barriers to Ionization (*E*_b_^I^) and Trapping (*E*_b_^ST^) of Hole Polarons for Different Phases: Anatase (An), Brookite (Br), and TiO_2_-B (B)

site	*E*_b_^I^ (eV)	*E*_b_^ST^ (eV)
O^An^	0.24	0.03
O_1_^Br^	0.12	0.05
O_2_^Br^	0.10	0.07
O_1_^B^	0.42	0.00
O_2_^B^	0.53	0.00

The reported values for ionization and trapping have strong implications in p-type doping for semiconductors. For example, in TiO_2_-B, we can see that there is not only a relatively high barrier to ionization, but there is no barrier to trapping. There remains no barrier to trapping even when the spacing between points in configuration-coordinate space is reduced to 0.01Δ*Q* in the region of interest. This means that TiO_2_-B would be difficult to dope p-type. The opposite can be said for brookite as the ionization barrier is much lower, while the trapping barrier is higher, suggesting it could be accessible for p-type doping.

### 4.3 Long-Range Polaronic Diffusion in Anatase, Brookite, and TiO_2_-B

Comparison of the calculated barriers to thermal ionization of hole polarons into the valence band against the calculated barriers for hole hopping allows one to make immediate conclusions on the mechanism of hole transport in the three phases of TiO_2_ considered. First, as noted above, the barrier to thermally ionize hole polarons in brookite is very small (0.12 eV) and almost all hole hopping pathways have larger activation energies. The barrier to self-trapping is also in comparison large (0.05 eV). This suggests that the most likely mechanism for long-range hole migration in brookite is thermal ionization into the valence band, followed by band-like conduction and subsequent retrapping. Hence, we predict brookite should be associated with the highest hole mobility of the three phases considered.

**Figure 5:**
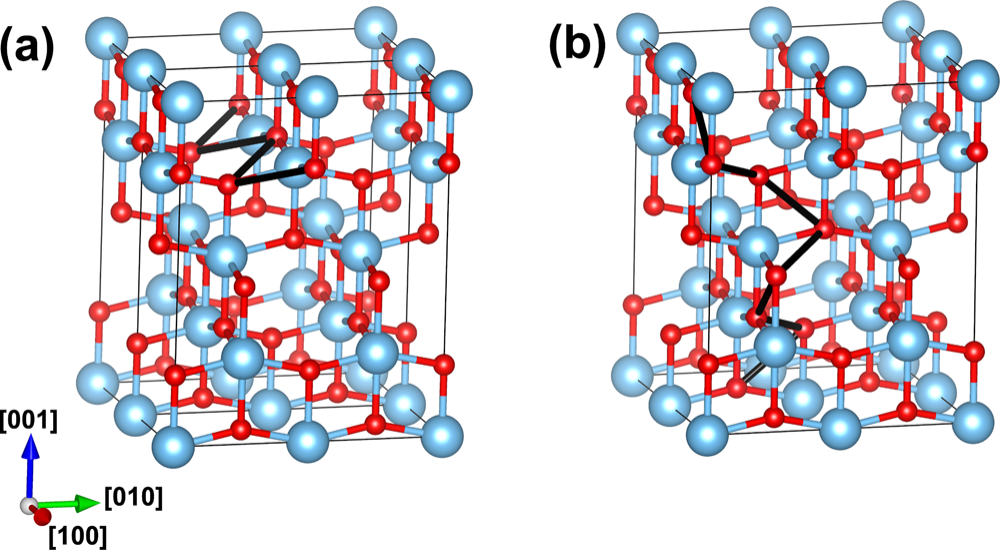
(a) Most favorable pathway for long-range diffusion of holes in the [100] or [010] directions in anatase with a rate-determining activation barrier of 0.12 eV. (b) Most favorable pathway for long-range diffusion of holes in the [001] direction in anatase with a rate-determining activation barrier of 0.20 eV.

While the barrier to thermal ionization of hole polarons in anatase is still relatively small (0.24 eV), several pathways for hole hopping have smaller activation energies. Long-range diffusion of hole polarons in the [100] and [010] directions is possible by constructing diffusion paths consisting of the most favorable pathway (i), which has an activation energy of only 0.12 eV. An example of such a path is shown in Figure 5a. Long-range diffusion in the [001] direction requires combining elementary pathways of types (i) and (ii) (Figure 5b); hence, the activation barrier for the rate-limiting step in this case is 0.20 eV. The similarity of activation energies for ionization and hopping suggests that long-range hole migration in anatase may have a mixed character. At a very low temperature, the dominant process would be hole hopping, which would be highly anisotropic with diffusion in the [100] and [010] directions preferred. At higher temperatures, one would expect a mixture of hole hopping and thermal ionization/retrapping resulting in an intermediate hole mobility. We discuss this in more detail in the subsequent section.

**Figure 6:**
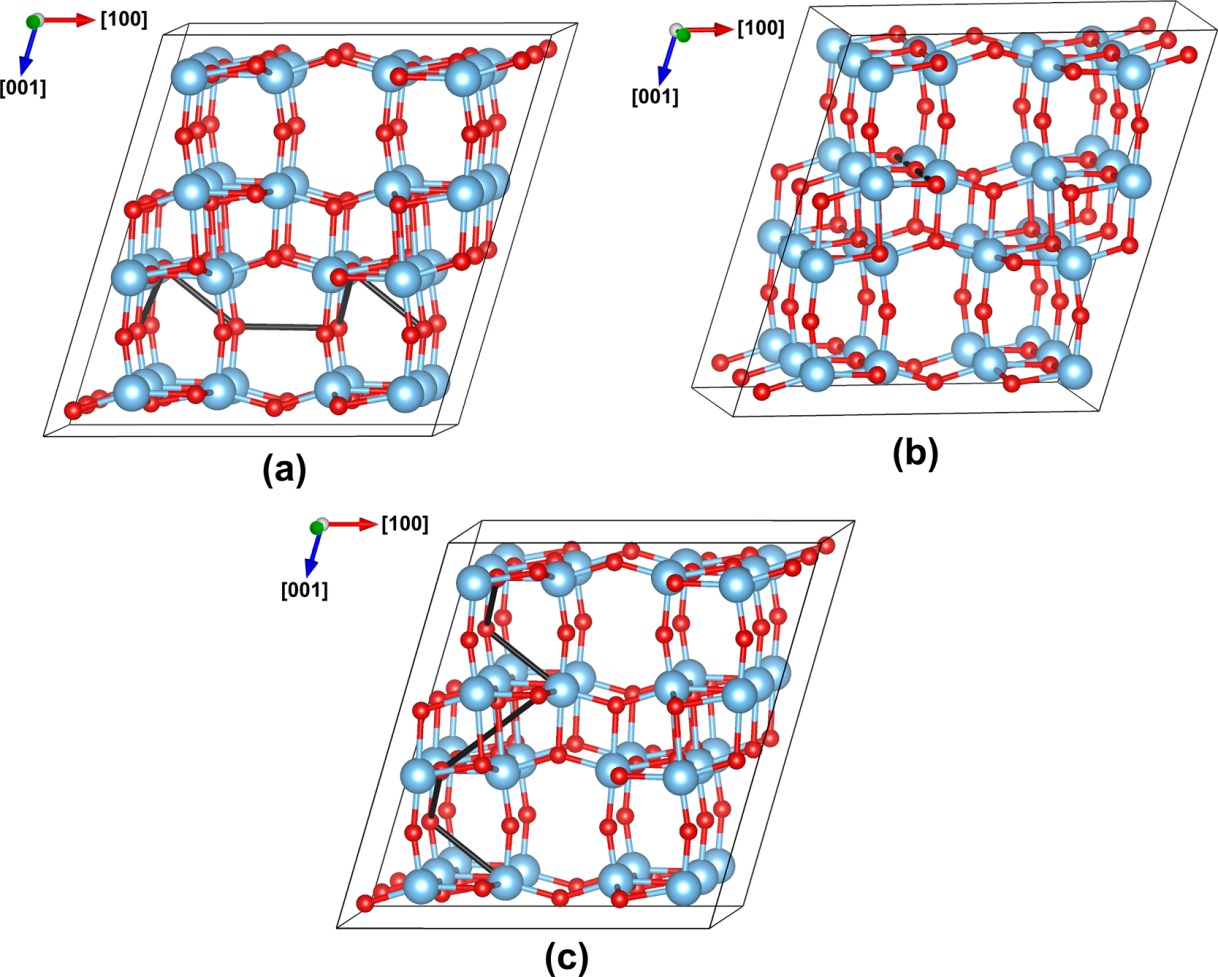
Most favorable pathways for long-range diffusion of holes in TiO_2_-B in the (a) [100], (b) [010], and (c) [001] directions, with rate-determining activation barriers of 0.39, 0.39, and 0.41 eV, respectively.

The barrier to thermal ionization of hole polarons in TiO_2_-B is much larger (0.53 eV) and although activation energies for hole hopping are also large, they are all smaller than the barrier to ionization. As for anatase, one can again determine favorable pathways for long-range hole diffusion in each crystallographic direction and corresponding activation energies for the rate-limiting elementary steps. As shown in Figure 6a, the most favorable path for diffusion in the [100] direction is for holes to hop from the O_1_ site to the less stable O_2_ site where barriers to intersite hopping are lower. The elementary paths involved are (i), (ii), and (v) with a rate-determining activation barrier of 0.39 eV. Diffusion in the [010] direction can proceed directly between O_1_ sites via pathway (vi) with an activation energy of 0.39 eV (Figure 6b). Finally, diffusion in the [001] direction involves elementary paths (i), (ii), and (vii), with a rate-determining activation barrier of 0.41 eV (Figure 6c). Since there is no barrier to hole self-trapping in TiO_2_-B and the barrier to thermal ionization is large, one expects hole polarons will readily form. As for anatase, the rate-determining activation barriers for long-range hole hopping are slightly smaller but comparable to the barrier for thermal ionization and so one expects migration to have a mixed character. However, the activation energies involved are much higher than for anatase and so TiO_2_-B is predicted to have the lowest hole mobility of the three phases.

## 5 Discussion

Quantitative prediction of hole mobility would require a full kinetic Monte Carlo simulation; however, one can provide order of magnitude estimates using the most favorable diffusion pathways we have determined together with the Einstein relation (e.g., see ref (26)). In anatase, at room temperature, the mobility associated with hole hopping between adjacent sites is of the order 10^–3^ cm^2^/V s while the ratio of hopping events to ionization events is around 10^2^. Since the typical diffusion length of a band-like carrier could easily exceed that achieved by 10^2^ hops between adjacent sites, this suggests that hole mobility at room temperature in anatase has a significantly mixed character (i.e., both ionization/retrapping and small polaron hopping). At nitrogen temperatures (77 K), hopping events are around 10^8^ times more probable than ionization. Therefore, in this regime, mobility should be dominated by small polaron hopping and exhibit significant anisotropy. Since TiO_2_ is usually intrinsically n-type, information on hole mobilities is scarce, particularly for the less common phases. The review by Bak et al. surveys some of the data available in the literature, including single-crystal and polycrystalline materials.^65^ However, direct comparison to experimental data is challenging since it convolutes the intrinsic polaron mobility in the bulk material with effects of point and extended defects which are not easy to disentangle. Nevertheless, our estimates of mobility are comparable in magnitude to reported experimental mobilities and previous theoretical calculations.^26,65^ The prediction that anatase should exhibit a mixed character for hole polaron mobility is consistent with time-resolved photoluminescence, photoconductance, and transient absorption spectroscopy studies of anatase single crystals, which attributed non-exponential decays (not seen in rutile) to the presence of multiple carrier trapping processes.^66^

There is very little existing data for brookite or TiO_2_-B but our calculations suggest very distinct behavior that could be probed by electrical conductivity measurements. Hole mobility in brookite is predicted to be the highest of the three phases and involve thermal ionization from small polaron configurations followed by long-range band-like diffusion and retrapping. On the other hand, hole mobility in TiO_2_-B is associated with very high activation energies (for both hopping and ionization). At room temperature, the small polaron hopping mobility is of the order 10^–7^ cm^2^/V s with a ratio of hopping to ionization of around 10^2^. Therefore, as for anatase, we predict a mixed character of mobility at room temperature. At nitrogen temperatures, small polaron hopping again dominates, being around 10^10^ times more probable than ionization, but unlike anatase, we predict fairly isotropic mobility.

The results discussed above have implications for applications on TiO_2_ in photocatalysis and photoelectrochemistry. Equilibrium anatase nanocrystals expose predominantly {101} surface facets. Since the most rapid diffusion pathways in anatase are along the [010] and [100] directions, we predict holes that are photogenerated in the bulk of the nanocrystal will readily migrate to the {101} surfaces where they may interact with adsorbates and facilitate reactions.^31^ Diffusion to {001} facets on the other hand would be less facile, particularly at lower temperatures, where the mobility takes on a stronger hopping character. For TiO_2_-B, we expect much reduced hole mobility in general and migration of holes to surfaces of nanocrystals will be much less efficient than for the other phases. This would suggest that TiO_2_-B nanocrystals are less active toward photo-oxidation than the other phases.

We conclude this section with a discussion of the factors that may affect the accuracy of the predictions. One of the largest potential sources of inaccuracy is residual SIE that can lead to inaccurate results for the stability and localization of small polarons. Our approach is explicitly parameterized to minimize SIE, with linearity of the total energy with respect to fractional occupation satisfied to less than 0.05 eV.^14^ We have also employed comparatively large supercells in our calculations, which should minimize undesirable finite size effects. The one-dimensional model for electron transfer we employ in conjunction with MEHAM theory are more significant simplifications. More general approaches are available but come with increased computational costs.^67^ While quantitative prediction of rates should not expected, this simpler approach should be sufficient for identifying the most favorable pathways for migration and order of magnitude estimates of electron hopping rates.

## 6 Conclusions

In this study, we have used hybrid DFT that has been explicitly parameterized to minimize self-interaction errors (ensuring accurate description of charge-carrier trapping) to model hole polaron migration, ionization, and trapping in the anatase, brookite, and TiO_2_-B phases of TiO_2_. We find that the hole polaron mobility increases in the order TiO_2_-B < anatase < brookite. There are also distinct differences in the character of hole polaron migration in each phase. In brookite, hole diffusion proceeds via thermal ionization of the small polaron, band-like diffusion of the hole, and subsequent retrapping. At room temperature, hole polaron diffusion in anatase and TiO_2_-B has a mixed character (combining short hops between neighboring oxygen sites with less frequent thermal ionization and retrapping). At nitrogen temperatures, the migration character in both these materials becomes polaron hopping-dominated, with anatase exhibiting significant anisotropy. The activation energies for polaron ionization and hopping in TiO_2_-B are very high (around 0.4–0.5 eV), suggesting very poor hole mobility. These predictions should serve as a guide for future experiments aiming to probe charge-carrier dynamics in TiO_2_, a very challenging problem due to the need to disentangle a number of effects due to point and extended defects. As well as having fundamental importance, we have discussed the implications for applications of TiO_2_ in photocatalysis and photoelectrochemistry.
